# The Use of Deep Learning Methods for Object Height Estimation in High Resolution Satellite Images

**DOI:** 10.3390/s23198162

**Published:** 2023-09-29

**Authors:** Szymon Glinka, Jarosław Bajer, Damian Wierzbicki, Kinga Karwowska, Michal Kedzierski

**Affiliations:** 1 Creotech Instruments S.A., 05-500 Piaseczno, Poland; jaroslaw.bajer@creotech.pl; 2 Department of Imagery Intelligence, Faculty of Civil Engineering and Geodesy, Military University of Technology, 00-908 Warsaw, Poland; damian.wierzbicki@wat.edu.pl (D.W.); kinga.karwowska@wat.edu.pl (K.K.); michal.kedzierski@wat.edu.pl (M.K.)

**Keywords:** remote sensing, satellite imagery, height estimation, segmentation, deep learning, earth observation

## Abstract

Processing single high-resolution satellite images may provide a lot of important information about the urban landscape or other applications related to the inventory of high-altitude objects. Unfortunately, the direct extraction of specific features from single satellite scenes can be difficult. However, the appropriate use of advanced processing methods based on deep learning algorithms allows us to obtain valuable information from these images. The height of buildings, for example, may be determined based on the extraction of shadows from an image and taking into account other metadata, e.g., the sun elevation angle and satellite azimuth angle. Classic methods of processing satellite imagery based on thresholding or simple segmentation are not sufficient because, in most cases, satellite scenes are not spectrally heterogenous. Therefore, the use of classical shadow detection methods is difficult. The authors of this article explore the possibility of using high-resolution optical satellite data to develop a universal algorithm for a fully automated estimation of object heights within the land cover by calculating the length of the shadow of each founded object. Finally, a set of algorithms allowing for a fully automatic detection of objects and shadows from satellite and aerial imagery and an iterative analysis of the relationships between them to calculate the heights of typical objects (such as buildings) and atypical objects (such as wind turbines) is proposed. The city of Warsaw (Poland) was used as the test area. LiDAR data were adopted as the reference measurement. As a result of final analyses based on measurements from several hundred thousand objects, the global accuracy obtained was ±4.66 m.

## 1. Introduction

The development and availability of Earth Observation (EO) data generate enormous possibilities for analyzing the Earth’s surface from various perspectives, starting from macro-level analyses concerning the occurrence of phenomena or changes over large areas of land, such as land cover [1,2], land irrigation, and monitoring the state of water reservoirs [3], to micro-level analyses enabling the detection of individual objects [4] such as ships [5] or airplanes [6]. Furthermore, EO data often capture phenomena that are not visible when using conventional methods, thanks to the registration of a broader spectrum of electromagnetic radiation. This allows for the utilization of such data, particularly in precision agriculture [7] or the detection of detailed environmental changes [8,9], as well as supporting actions related to managing urban areas [10] or the management of natural hazards [11].

This article primarily analyzes EO data obtained from satellites orbiting the Earth. Data acquired from this altitude may be categorized based on the type of sensor used and its quality. Among the data, one may primarily distinguish those acquired by passive sensors (mainly optical sensors) and active sensors (such as those recording microwave bands). The characteristics of products from different sensors, including spatial, radiometric, and temporal resolution, may vary and are often dependent on the data distribution method, whether commercial (such as the WorldView or Pleiades missions) or open (e.g., the Landsat or Sentinel missions).

Optical satellite imagery provides a wealth of information regarding land cover. Especially when utilizing multi-spectral sensors, it can be used for land surface observation, terrain mapping, environmental monitoring, atmospheric studies, detecting landscape changes, monitoring natural disasters, and resource management. Optical satellite data allow for the assessment of vegetation changes, air pollution, water quality, forest degradation, soil erosion, and other environmental hazards. They also support environmental protection and sustainable development efforts [12]. On the other hand, these data are often limited by atmospheric factors, which may hinder their usability within pre-defined time periods.

The increasing volume of EO data from satellites requires appropriate processing and the development of the relevant applications. The challenges associated with this primarily involve creating algorithms for specific purposes. This is also driven by the need for greater computational resources, in particular efficient solutions based on Graphics Processing Units (GPUs), which are particularly useful for creating machine learning-based algorithms.

Based on the available data and developed or modified existing algorithms, the authors decided to explore the possibility of using high-resolution optical satellite data to develop a universal algorithm for a fully automated estimation of object heights within the land cover. For the purposes of this article, the term “land cover objects” is understood as both anthropogenic and natural features that protrude above the ground surface. The existing literature mainly focuses on buildings and lacks full automation [13,14,15]. Thus, the solution presented here undoubtedly brings novelty. In summary, this article investigates the possibility of creating a comprehensive, fully automated solution that utilizes deep neural network models to extract information from imagery (artificial objects and shadows); this is also the purpose of this article. The authors would also like to emphasize that this paper focuses on a holistic description of an algorithm for calculating the height of objects from single remote sensing images, without discussing details of, for example, the datasets created or architectures used. These may be the subject of future publications and are only briefly described in this article.

To address these challenges, this study presents a novel approach to estimating the height of land cover in high-resolution satellite images by analyzing one image at a time. There is a strict correlation between the resulting lengths of shadows and the angles of a sensor. Acquisition metadata help us understand the geometric correlation between buildings and shadows in various situations, and thus to calculate their exact height. The main goal was to create an algorithm that would use shadow segmentation and building segmentation to automatically calculate the height of a building in a pre-defined area. With automatic processing and well-trained models, it is possible to obtain precise results and create novel ideas that will use this kind of data, e.g., for monitoring obstacles near airports.

The structure of this article is as follows: In the second chapter, the research background and related works are presented. The third chapter introduces the research area, the materials used, and the research methodology. The fourth chapter presents the results, including a description of the algorithm calibration process. The fifth chapter contains a discussion of the results, along with a description of the limitations of the obtained outcomes. This article concludes with a summary of the research findings and a presentation of the overall conclusions of this study.

## 2. Background and Related Works

### 2.1. Geospatial Data

Geospatial data resources are expanding practically every day, yet their widespread utilization is still not common. While the number of applications built upon these resources continues to increase, it still seems that their full potential is not being fully realized [16].

Open geospatial data resources may be divided based on their origin and type. They may also be collected and shared either globally or at the local level (e.g., by national mapping agencies) following specific data opening strategies governed by regulations, such as those introduced by the European Union (e.g., INSPIRE). These strategies provide quick, structured, and easy access to data collected by national mapping agencies. These data include vector data that represent buildings in a given area from cadastral resources, raster data such as orthophotos, or LiDAR point clouds. On the other hand, globally available data allow for macro-level analysis. Notable examples include missions like Landsat and Sentinel, as well as platforms like EarthExplorer, which offer a wide range of products (e.g., SRTM), and volunteer-based services like OpenStreetMap (OSM) [17].

Furthermore, the amount of data offered by commercial providers is continuously increasing. Satellite missions from companies like Maxar and Airbus provide products with spatial resolutions as fine as 0.30 m, including stereo imagery for generating elevation data. Similarly, SAR data provided by companies like ICEYE also feature high spatial resolution. The spatial resolution offered by the newest satellites may be as fine as 1 m on the ground in multi-looked images. Azimuth resolution may even reach 5 cm, and range resolution may reach up to 50 cm, depending on the acquisition mode.

A detailed description of individual satellite missions acquiring geospatial data, often accompanied by data quality assessments, can be found in [18].

### 2.2. Deep Learning for Geospatial Data

Deep learning solutions for geospatial data are becoming increasingly diverse. This applies to tasks related to image segmentation, such as creating land cover maps or segmenting specific objects like buildings [19], as well as object detection tasks [5].

Taking a holistic approach to such data and algorithm development, it is important to acknowledge their diversity. This applies to the spatial, radiometric, and qualitative characteristics of individual data. These diversities pose challenges in developing universal algorithms and constructing appropriate datasets. For example, variations in data representation can arise from different imaging times or orientation parameters [20]. However, these challenges can be addressed with the advancement of algorithms and methods for automatic dataset generation, such as those demonstrated in [21]. Additionally, the ability to train algorithms that are less specific to a particular problem, such as those based on transformers [22], offers further potential in overcoming these obstacles.

In exploring deep learning solutions for geospatial data, our focus will primarily be on the tasks outlined in the project, namely shadow segmentation and building segmentation. However, it should be noted that the goal of the algorithm development was not to create state-of-the-art solutions but rather to utilize existing methods and implement them from scratch. Additionally, the datasets created for training the algorithms will only be partially described.

When it comes to building segmentation, it is one of the most common tasks in the context of analyzing data acquired from satellite or aerial platforms. The applications of these solutions vary, ranging from verifying cadastral data [19] to automatically mapping buildings in unmapped areas. A comprehensive review of such solutions was conducted in [23]. The main challenges in this field include generating datasets, utilizing various architectures to improve the accuracy of the defined task, optimizing hyperparameters (e.g., loss functions), and using post-processing techniques to achieve the desired vector representation.

Looking specifically at deep learning solutions for building segmentation, the utilized architectures primarily include various variants of UNet [24,25], DeepLabV3+ [19], HRNET [26], and GAN-based approaches [27], as well as those based on transformers [28]. These architectures have commonly been employed to tackle the task of building segmentation and have shown promising results in extracting accurate building footprints from satellite or aerial imagery.

Shadow segmentation is a relatively common problem in computer vision, but it is typically addressed in the context of regular images rather than remote sensing. As far as remote sensing imagery is concerned, shadows can be considered as something that interferes with tasks such as calculating remote sensing indices and requires removal or replacement [29]. However, as in the present publication, shadow segmentation may also be utilized to extract valuable information from images. In this context, the goal is to accurately delineate and analyze shadow areas for specific applications.

Shadow detection and segmentation for remote sensing data can be achieved using both classical computer vision methods and deep neural networks. Among the classical methods, the utilization of shadow indices [29], Principal Component Analysis (PCA) [30], or ghost image-based approaches [31] can be distinguished. When it comes to deep learning solutions, commonly used architectures such as UNet and DeepLab [32] are employed, as well as structures that incorporate global–local awareness for a more effective feature fusion at both the local and global levels [33], or models that focus on analyzing contextual information [34]. These deep learning approaches aim to improve the accuracy and robustness of shadow detection and segmentation tasks, leveraging the power of neural networks to handle complex image characteristics and variations in shadow appearance.

In creating the solution presented here, the authors focused on creating the best possible dataset to train network models that would be capable of detecting most objects located above the ground surface. To create such a dataset, automated data labeling and broad access to geospatial data through an open API were used to enable the generation of high-quality training and validation datasets. This process was carried out similarly for both shadow detection and building segmentation. Thanks to carefully selected network architecture parameters, a reliable and fully automatic solution that combines image processing, machine learning, and data science was developed successfully. Therefore, this solution stands out for its repeatability and unique approach, combining machine learning techniques in parallel with classical image processing and database integration, distinguishing it from other methods presented earlier in this chapter.

### 2.3. Height Estimation

**Figure 1:**
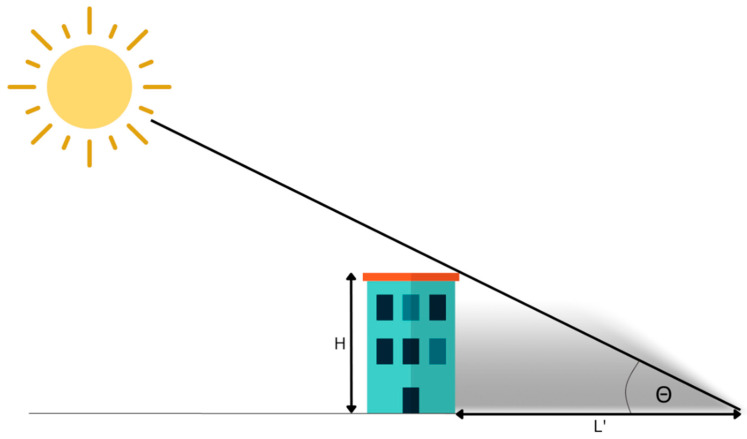
The relationship between the length of the shadow (L′) of an object, elevation angle of the sun (Θ), and building base height (H) (Equation (1)).

Taking both the direct content of Earth Observation (EO) data, which is the pixel-level representation, and the indirect content, which consists of the metadata associated with the imagery, allows us to perceive the potential of extracting valuable information from the data. By performing appropriate measurements on the image, locating specific information in the image metadata, integrating these data sources, and subsequently conducting simple calculations based on the aggregated data, highly valuable insights regarding the parameters of objects present in the imagery may be derived [35,36]. This holistic approach enables us to extract meaningful knowledge and valuable geospatial information from EO data. Basing on simple trigonometric functions and the geometry of the Sun–satellite–object system (three-element configuration), it is possible to establish a simplified relationship between the length of the shadow cast by a specific object, as captured and measured in a single satellite image, and the height of that object. The starting point for the mathematical description of the three-element configuration defined in the above paragraph are the relationships occurring in the Sun–object system, which are illustrated in Figure 1 and represented by Equation (1).

(1) $$L^{\prime }=\frac{H}{tan(\theta )}$$

The main problem that arises is the influence of the sensor’s position, often resulting in the inability to obtain a complete view of the shadow of a given object [36]. This issue becomes particularly significant when there is no access to data that would allow for the detailed positioning of the vector on the ground. In such cases, corrective operations are required to adjust the length of the shadow according to the algorithm described in [13,15,35]. The individual shadow parameters are then calculated based on an iterative approach that utilizes the orientation parameters of the sensor and the sun.

In the investigated case, the acquired vector data represent the footprints of objects, and this issue can largely be overlooked. However, it requires aligning the shadow with the vector, taking into account the different signs depending on the satellite’s azimuth. The situation is different, for example, for objects obtained by segmentation, which may be affected by radial displacements. The resulting vector corresponds to the roof of the object, and for such cases, the correction of occlusion or excessive shadow exposure should be taken into account. The complex estimation method presented by V.K. Shettigara and G. Sumerling [35] is a significantly more accurate approach for determining object height compared to the simple method. Under favorable computational conditions (i.e., when the measurements of shadow surface, length, and range, as well as object azimuth, are taken from high-resolution imagery), the complex method can serve as a viable means of determining object height.

Taking a holistic view of solutions for estimating object heights based on EO data, various approaches can be mentioned, considering both the analyzed measurement product and the type of sensor used. In the literature, solutions based on the sensor–Sun–object relationship can be found, as well as those analyzing depth information or calculating the radial displacements of the objects in the image. Attempts to determine heights are carried out for imagery in the optical spectrum acquired from aerial or satellite platforms, as well as for imagery in the radar spectrum.

Solutions based on attempting to determine heights directly from individual images employ an approach based on monocular vision (depth), which aims to analyze the depth information in the image, often using deep machine learning techniques. This approach allows for the generation of normalized Digital Surface Models (nDSM), as demonstrated, for example, in the case of orthophotomaps in [37], although tall objects (above 100 m) were not considered. Similar approaches were applied in [38,39,40], where the authors utilized encoder–decoder networks to create height maps. A slightly different approach was taken by the authors of [41], who also incorporated the idea of style transfer. Considering the characteristics of satellite data (lower resolution and greater diversity compared to the imagery analyzed in the publications), as well as the limitations of the existing solutions discussed in these publications, it was decided to utilize the approaches described further.

Another group of solutions involves non-optical sensors or the use of multiple images. While the literature provides numerous references for solutions for data acquired from aerial platforms based on classical photogrammetric methods, the scope of this article primarily focuses on satellite data. In this case, height estimation is performed by measuring pairs of optical images. The authors of [42] proposed the use of an EDM (Building–Ground Elevation Difference Model) for extracting the ground level and building heights. A comparison between backward and forward images was also conducted in [43]. These authors also proposed a manual algorithm that allows for an iterative approach to extract elements on building rooftops. Another solution based on these ideas is presented in [44].

Indeed, height estimation attempts have also been made based on SAR data. However, due to the nature of SAR, these methods often require the use of projected heights [45]. Despite this limitation, SAR data allow for accurate height estimation, even in mountainous areas [46]. The specific characteristics of SAR data make them a valuable source for height estimation, particularly in areas where optical data may be limited or less effective.

The final group comprises solutions similar to the proposed approach, but they involve non-automated methods or focus on specific types of objects. Some of these studies concentrate on shadow detection based on image operations and then calculate the heights of specific buildings using the elevation angle in a single iteration [13]. In [47], an inclination correction algorithm is proposed to improve the accuracy of building height estimation. An approach based on shadow overlapping is applied in [48], where shadow reduction for vegetation using the NDVI index is utilized to remove vegetation near objects. Analyses and methods for different scenarios, such as urban and mountainous areas, are presented in [49]. In [50], a method based on shadow analysis using Faster R-CNN and image metadata is proposed. An automated method called Volumetric Shadow Analysis (VSA) is also suggested in [14].

Our method combines several of the aforementioned approaches to achieve the best possible results and is comprehensive in nature, not focusing solely on buildings but also on other objects.

## 3. Methodology

### 3.1. Materials

During the implementation of tasks related to algorithm development, a wide range of publicly available solutions described in the previous section were utilized. These include primarily the described architectures of deep neural network models, as well as algorithms for post-processing and for the height estimation of objects from single images.

Furthermore, during the process of training the deep neural network models, a computer with the following specifications was used: NVIDIA Quadro P5000 GPU, 2 × Intel XEON Gold 5220R CPUs, and 256 GB RAM.

During the algorithm development process, the Python programming language was primarily used along with a wide range of geospatial analysis libraries such as rasterio, GDAL, geopandas, as well as communication with PostgreSQL databases using the PostGIS extension. The TensorFlow library was utilized for building and training the neural network models. The input data for the algorithm were standardized and followed the GeoTIFF format.

### 3.2. Study Area

Research was primarily conducted in the area of Warsaw, the capital of Poland, due to the availability of relevant data for this region and its diverse characteristics, including densely built urban areas as well as forested regions. However, it should be noted that the datasets created for machine learning methods during the research were generated based on a much broader area encompassing other regions in Poland and worldwide. When their quality was suitable, open data sources were also utilized after proper verification. The data were divided based on the type of task and process, including training, validation, and testing.

**Figure 2:**
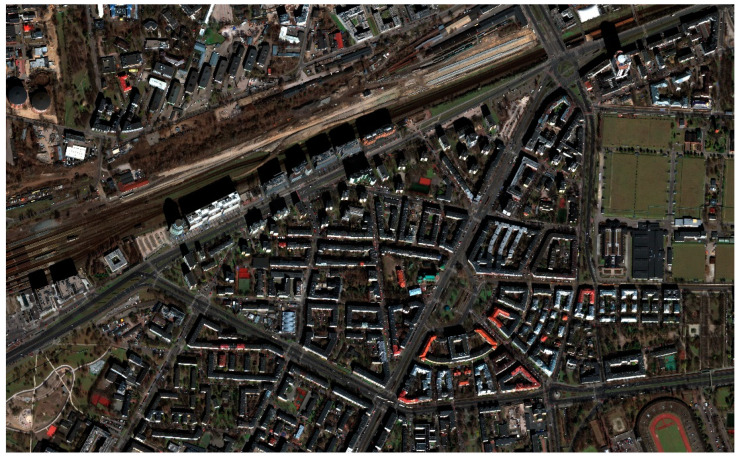
A sample image covering the analyzed area (Warsaw, Poland).

A sample image covering the analyzed area is shown in Figure 2.

The data used for the final analysis of the Warsaw area were obtained from the DigitalGlobe mission—WorldView-3, with an acquisition date of 5 April 2020. As reference data for comparison with the final results, laser scanning data provided by the Polish national mapping agency (GUGiK) through its geoportal [51] were used. The data covered approximately 300 square kilometers.

### 3.3. Pipeline Schema and Algorithm Description

**Figure 3:**
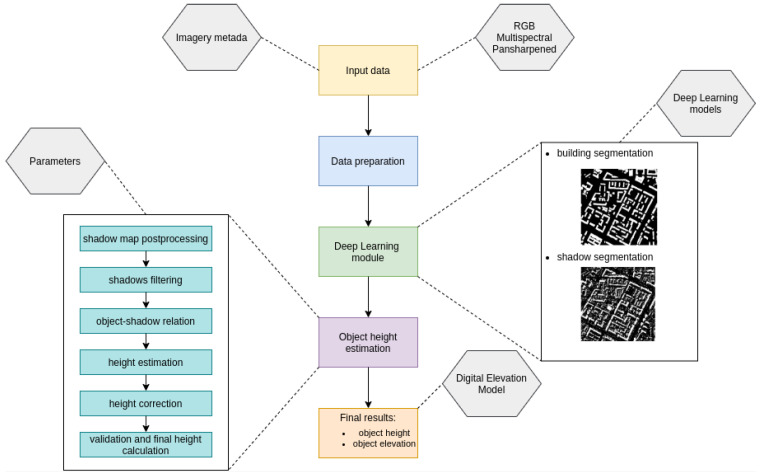
Methodology and pipeline schema.

The algorithm begins its operation by loading the data (Figure 3). There are various configurations possible, but they require additional metadata to describe the input image. The metadata should include information that allows for the direct or indirect interpretation of the sensor’s position during image acquisition, as well as the position of the sun. For the purposes of this study, position refers to elevation angle, azimuth, and image extent. These metadata are usually provided for satellite imagery. As for the sun, it is also possible to determine its position based on the location of the imaging and the date. Additionally, if necessary for georeferencing improvement, the image may contain information about transformation parameters based on Ground Control Points (GCPs).

Importantly, the presented algorithm is designed to be universal, meaning that it should support both high-resolution satellite imagery and conventional RGB imagery, such as orthophotos. However, it is important to note that additional data, as described in the previous paragraph, may be required to process the images accurately.

The next step is data preparation. At this stage, various processes are applied depending on the data type, such as georeferencing correction through transformation if Ground Control Points (GCPs) are available. Additionally, sharpening or file validation processes may be performed. Following that, normalization takes place. Due to the aim of universality and handling different data types, each processed image is saved using 8 bits for each of the three channels: Red, Green, and Blue. This approach poses a challenge since it often results in information loss for satellite imagery. However, it allows for a universal approach. Image normalization can be done in various ways. In our case, the authors chose to use a simple linear normalization method, which involves clipping the lowest and highest percentile values and assigning values from 0 to 255 to the remaining pixels based on the histogram distribution.

The next stage involves utilizing Deep Neural Networks (DNNs) to extract specific information from the imagery. Here, the main focus are two aspects: buildings and object shadows. During the early stages of algorithm development, the authors experimented with PCA- and GAN-based approaches for shadow segmentation, but they did not yield satisfactory results (this analysis will be omitted and can be the subject of a future article). Throughout the development process, various architectures were tested, including HRNETv2, DeepLabV3+, different variants of UNet, and transformer-based models. However, it was ultimately decided to use a modified version of the UNet model, which incorporates skip connections and includes a channel spatial attention module with a ResNetV2-101 backbone. The same model architecture was applied for both building extraction and shadow detection. Additionally, a combination of boundary-aware loss and weighted binary cross-entropy dice loss was used to accurately reconstruct the positions of both buildings and shadows.

The database of objects obtained through machine learning was supplemented with objects available in open resources (described in Section 2.1). For this purpose, algorithms were developed to effectively retrieve objects from global databases such as OSM (OpenStreetMap) and local databases related to topographic objects. Algorithms for retrieving data were developed based on existing libraries such as osmnx (for retrieving OSM data), or methods were created to retrieve data from local databases provided by the NMAs (National Mapping Agency) of individual countries using APIs or WMS or WFS services. These algorithms enable the efficient extraction of objects and their integration into the final dataset.

Then, objects that are completely in shadow, such as smaller objects near a tall object, are discarded. Additionally, a shadow map correction is performed based on the objects. Subsequently, morphological operations are applied to refine the shadows. Dilation with a 3 × 3 kernel size is primarily used to close the holes in the shadow map often caused by objects such as bright cars. These operations help to enhance the accuracy and quality of the shadow length estimation.

(2) $$H_{BASE}=\left(L_{BASE}+e\right)*tan(\theta )$$

where:

*H_BASE_*—base height of the object;
*L_BASE_*—estimated shadow length based on cutting line;
*e*—parameter calculated based on the lengthening or shortening of the cutting line (for vectors on the ground) or calculated in the correction process;
*θ*—elevation angle of the sun.

Next, cutting lines are created with a defined frequency, based on the reverse azimuth to the azimuth of the sun. The starting points of the lines are placed along the two edges of the image located on the sunny side. Frequency refers to the value of the spacing between each subsequent cutting line. In this study, in successive iterations, the best balance between the length of the processing time and the quality of the results was obtained when this parameter was set to 2.5 m.

In the next step, the cutting lines intersect with the shadow map to form shadow lines. For each line, the potential height of the object is calculated by multiplying the length of the shadow by the tangent of the sun’s elevation (based on Equation (1)). The shortest lines that are less than 5 m long are then dropped. This results in a reduction in the possible noise in the shadow map and significantly reduces computation time. Then, by applying a line extension (depending on the baseline shadow length) in the direction of the sun’s azimuth, a shadow line–object relationship is created based on the intersection. Once again, from the lines drawn for the object (shortened or extended depending on the position of the sensor), the height of the H_BASE_ object is calculated (based on Equation (2)). Thanks to this operation, for objects that are in the database (for which there is an existing vector on the ground), there is no need to carry out a correction due to the lack of visibility of the shadow at a later stage of the work. The parameter e is calculated based on the lengthening or shortening of the cutting line. An object can be associated with multiple heights, so using statistical methods based on percentiles and standard deviation, outliers are discarded, and the object is assigned the maximum value from the remaining heights. In the research phase, the best results were obtained for a value equal to the 90th percentile. This allowed for the removal of height values that could represent a potential arising error, for example, from the merging of several shadows into one. Furthermore, shadows for which no relationship with an object is found are validated, clustered, and assigned the class “unknown”.

In the next step, objects belonging to the “building” class are analyzed, and roof analysis is performed to add additional height elements to base height of the object *H_BASE_*. This is achieved by intersecting the translated building vectors based on the baseline height (due to radial shifts) with the shadow map. In the case of a vector derived from segmentation, this operation is not carried out because it represents an outline outside the roof or, in part, the roof and façade. Once again, cutting lines are created based on the reverse azimuth to the azimuth of the sun, along the edges of the buildings located on the sunny side. An intersection is then performed with the shadow map to create shadow lines. The maximum height value *h_roof_* is then determined (based on the shadow lines and Equation (1)) and added to the baseline height of the object. The final formula for buildings can therefore be rearranged as:

(3) $$H_{BUILDING}=H_{BASE}+h_{roof}$$

where:

*H_BUILDING_*—total value of the building height;
*H_BASE_*—base height of the object;
*h_roof_*—additional height value from the analysis of objects located on the roof.

This process, which was not commonly used before, allows for a more reliable estimation of the height of the buildings.

After the global analysis, a detailed analysis is conducted through the iteration of objects that require individual approaches. These include, e.g., power towers, wind turbines, and masts. Wind turbines, due to their characteristics and moving parts, require the calculation of the base height and the length of the blades. An approach that consists of calculating the base height based on the shadow from the turbine’s location to the intersection point of the blades (rotation center) determined by computer vision methods is implemented. Then, using existing catalogs, the blade length value is added to the baseline height.

Power towers and masts require individual approaches due to their often latticed structure, which makes it difficult to create a universal shadow model. To address this, a model was trained based on a modified UNet architecture specifically designed for segmenting lattice shadows. Using this model, the vertex of the lattice structure is identified in the direction opposite to the azimuth of the sun, allowing for the estimation of the object’s height.

**Figure 4:**
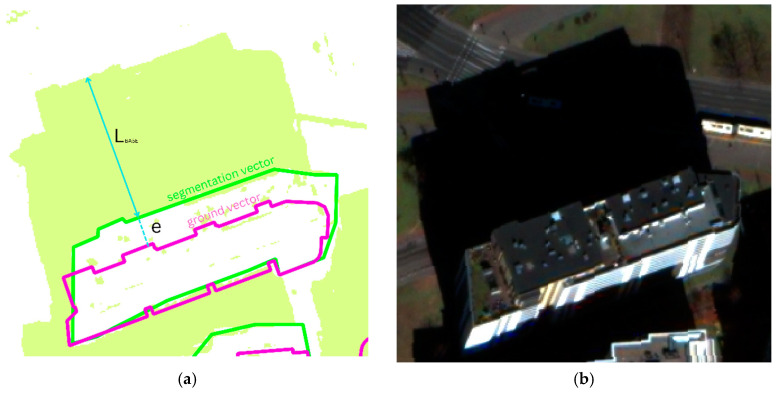
(**a**) Shadow length estimation—segmentation vector (green) versus ground vector (purple); light green color represents shadow. (**b**) Imagery fragment corresponding to (**a**).

After the extraction of information and calculation of object heights, the process of auto-validation and correction of results takes place, as described in the previous chapter. Auto-validation aims to detect and reject objects that have unrealistically assigned heights, such as trees. Therefore, the maximum parameters for the height attribute for each class were set to reduce the risk of gross errors. This may occur especially in the case of a shadow map problem and the merging of shadows of several objects. Furthermore, the correction process uses the formulae described in [13,15,35] to determine the value of e (2). The correction, however, was only applied to objects whose vectors did not represent an object on the ground, but were affected by a radial shift, e.g., a vector obtained through the use of segmentation. An example of this situation is presented in Figure 4. For objects that are represented by vectors at the ground level, correction may be applied due to the slope of the terrain on which the shadow is cast.

The obtained heights are relative values, so in order to give them a global character, a value is derived based on the Digital Elevation Model, which is acquired from local, national, or global resources (e.g., SRTM).

To effectively sort the results and perform an accurate analysis and auto-validation, a PostGIS database was used, enabling fast processing of geospatial queries on large collections of object data. Each object comes with certain attributes, including a classified class and the polygon of its outline.

Finally, vector layers representing individual objects along with their assigned class and height parameters are obtained. The schema representing the overall algorithm is included in Figure 3.

In summary, the entire process begins with the normalization and standardization of input data to ensure their consistent format and compliance with the algorithm’s requirements. By selecting Ground Control Points (GCPs), the imagery can be matched with data from open sources. Next, the data are tiled, and satellite imagery metadata are loaded to initiate shadow and building segmentation on each subsequent tile. Additionally, for atypical objects, a solution for detecting intermittent and less visible shadows is activated. Subsequently, the most appropriate line passing through the potentially longest shadow segment is determined, allowing for the calculation of the object’s height, coordinates, and outline using metadata. Finally, integration with a database takes place to enable the analysis of a large number of obstacles. The resulting solution operates on the principles of a data lake.

The main characteristics of the system are the following:

- Data Normalization and Unification: The system begins by normalizing and standardizing the input data to ensure consistency and compatibility with the algorithm’s requirements. This step is crucial for accurate processing and analysis.
- Ground Control Points (GCPs): The system utilizes GCPs to match satellite imagery with data from open sources. GCPs help establish spatial references and align different datasets, enabling reliable analysis.
- Tiling: The data are divided into tiles, which are smaller, manageable sections of the overall dataset. Tiling allows for the efficient processing and analysis of large quantities of geospatial data.
- Shadow and Building Segmentation: The system employs segmentation algorithms to detect and distinguish between shadows and buildings in satellite imagery. This enables the identification and measurement of objects above the ground.
- Handling Atypical Objects: The system includes a solution for detecting intermittent and less visible shadows, which helps to accurately identify and analyze atypical objects that might be challenging to detect using conventional methods.
- Height and Geospatial Data Calculation: The system calculates the height, coordinates, and outline of the detected objects using metadata and other relevant information from satellite imagery. This allows for a comprehensive understanding of the identified objects.
- Integration with Database: The system integrates with a database to store and analyze the results of the analysis. This enables the processing of a large volume of obstacles and facilitates data retrieval and querying.
- Data Lake Operation: The system operates based on the principles of a data lake. A data lake is a centralized repository that allows for the storage of structured and unstructured data. It provides a flexible and scalable architecture for data processing and analysis.

In summary, the system is characterized by its ability to handle large geospatial datasets, accurately detect and analyze objects above the ground, and integrate with a database for further analysis and decision-making. It combines various image processing, machine learning, and data science techniques to create a comprehensive and automated solution for geospatial analysis.

## 4. Results

### 4.1. Training, Validation and Assessment

#### 4.1.1. Training Process and Results

The training of models requires appropriate datasets. For both tasks, namely building segmentation and shadow detection, datasets were prepared. Both open resources, such as true orthophotos, and satellite imagery were used. Building vectors served as labels, obtained from local databases (e.g., cadastral data) as well as global sources like OSM.

For shadow detection, an iterative approach was employed, where shadow masks were first obtained using computer vision methods and then manually verified and corrected.

Additionally, available open datasets such as INRIA [52] were used to increase the number of images representing buildings, for example. The data, along with the masks, were divided into training, testing, and validation sets. The number of samples represented by 512 × 512-pixel images along with their masks is presented in the table below (Table 1).

The input data for the datasets covered various different regions of the world and Poland. These included images obtained at both the aerial and satellite levels. Aerial imagery was primarily from services provided locally (by the NMA), while global imagery came from our resources and included World-View 2, World-View 3, and Pleiades Neo mission imagery.

As mentioned in Section 3.3, various approaches and architectures were tested, but ultimately, a customized architecture based on UNet was utilized. This encoder–decoder architecture incorporated skip connections and a channel spatial attention element. A detailed analysis of the architecture might be the subject of a future publication as it yielded the best metrics for both building segmentation and shadow segmentation tasks.

The training process was the same for both tasks. It involved training for 50 epochs with a learning rate α = 0.001 and Adam optimizer parameters β_1_ = 0.9, β_2_ = 0.999, and ε = 10 − 7.

The accuracy of the trained network was evaluated using the mean Intersection over Union (IoU) metric. The Intersection over Union metric is calculated by finding the number of pixels where two objects overlap and dividing it by the total number of pixels covered by both objects.

(4) $$IoU=\frac{\mathrm{target}\cap \mathrm{detected}}{target\cup \mathrm{detected}}$$

The obtained mean Intersection over Union (IoU) metrics for the test set were 0.851 for the building segmentation task and 0.946 for the shadow segmentation task.

Below are the results of the model’s performance in various areas, as well as a comparison between the building prediction maps and vectors from open resources.

**Figure 5:**
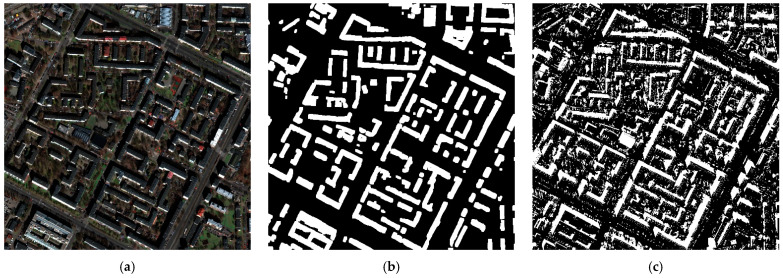
Example: (**a**) satellite imagery, (**b**) building mask prediction, (**c**) shadow map prediction.

**Figure 6:**
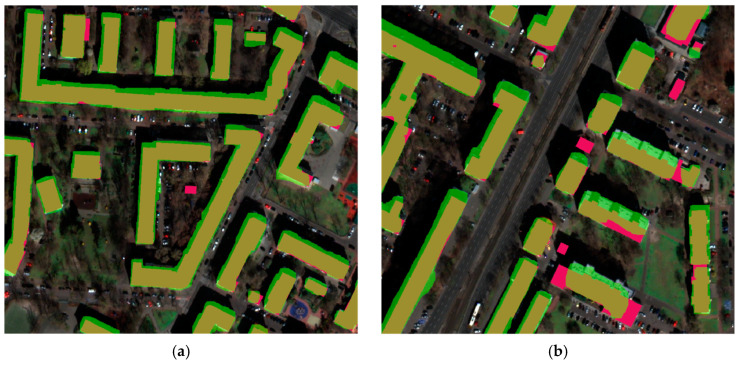
Building segmentation examples; (**a**) Example 1: green—building segmentation vector, (**b**) Example 2: pink—ground truth building vector.

The results obtained for building segmentation are presented in Figure 5 and Figure 6. In the first image, a generally satisfactory prediction outcome can be observed, despite the relatively dark input image. However, in the second image, certain issues stemming from image normalization or shadow effects can be noticed, which significantly affect prediction compared to the building vectors from OSM.

As the aim was to develop a universal algorithm that would be capable of conducting predictions on various images, of either aerial or satellite origin, the training data had to be adapted and normalized accordingly. The normalization process involved scaling the data from 0 to 255 and then further normalizing it to values ranging from 0 to 1 for the purpose of model training. This normalization procedure may result in the darkening or brightening of the images, which in turn can significantly impact predictions. This effect is particularly visible for objects with smaller footprints, those with more complex geometries, and those located in shadowed areas.

**Figure 7:**
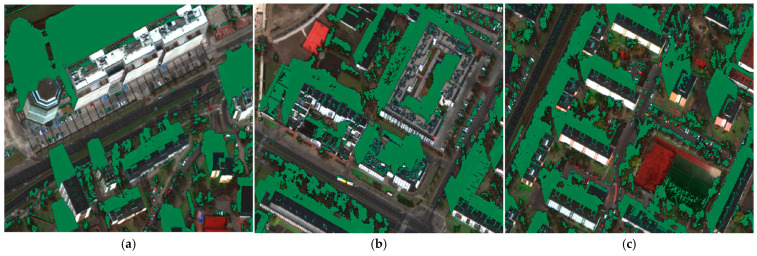
Shadow segmentation examples; green—recognized shadows. (**a**) Shadow detection in tall buildings. (**b**) Shadow detection in low buildings (**c**) Example of false positives—the area of artificial turf on the sports field shown.

The results obtained for shadow segmentation are presented in Figure 5 and Figure 7. The algorithm, as indicated by the mIoU metric, performed very well in terms of shadow extraction. It can be observed that the majority of shadows were identified accurately, including a detailed coverage of rooftops and shadow segmentation of vegetation. There were some isolated areas of False Positives (FPs) present, particularly for buildings with inclined roofs, where the representation differed slightly from the rest of the object, e.g., due to slightly smaller pixels. However, this issue was minimized during post-processing. Another example of FPs is the area of artificial turf on the sports field shown in Figure 7c. This can be attributed to spectral characteristics, light reflection, and normalization, where some of the darkest pixels were assigned RGB values close to zero, which could have led to the occurrence of FPs during prediction.

During the implementation of the algorithm, a problem was also identified with wetlands, water bodies, and water streams. This was due to the characteristic reflection of such objects, which are represented as dark areas that can be mistakenly interpreted as shadows by the algorithm. These objects were also eliminated during the post-processing stage to improve the accuracy of the segmentation results.

**Figure 8:**
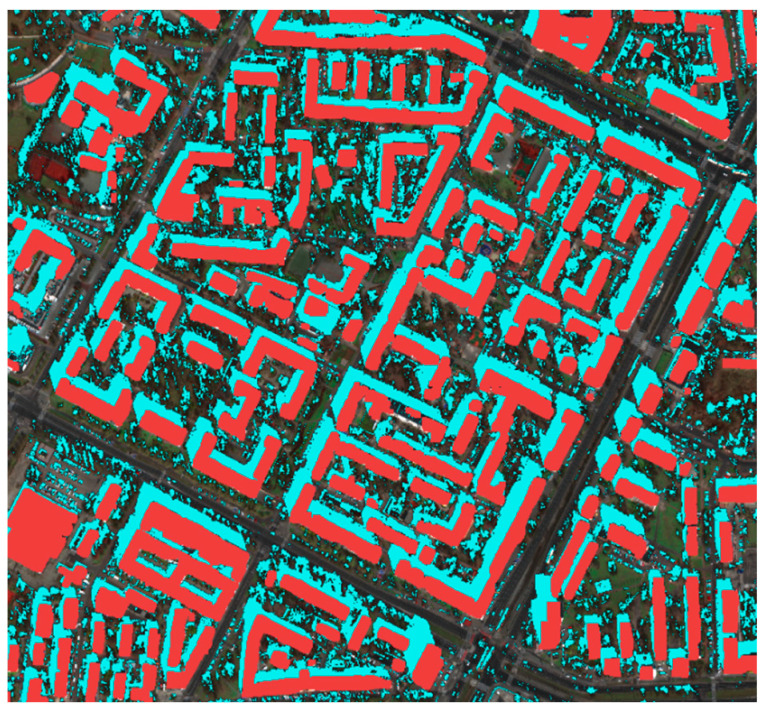
Joint visualization of buildings (red) and shadows (blue).

The overall results of the deep neural network models’ performance are presented in Figure 8.

#### 4.1.2. Height Estimation

This section presents the process of calculating the height of objects based on the method described in Section 3.3.

The input data for the calculations consist of vector representations of objects (from open databases as well as segmentation results) and a shadow map. In the initial phase, the shadow map undergoes pre-processing using visual morphological operations, and a vector representation of shadows is created to perform spatial analyses.

**Figure 9:**
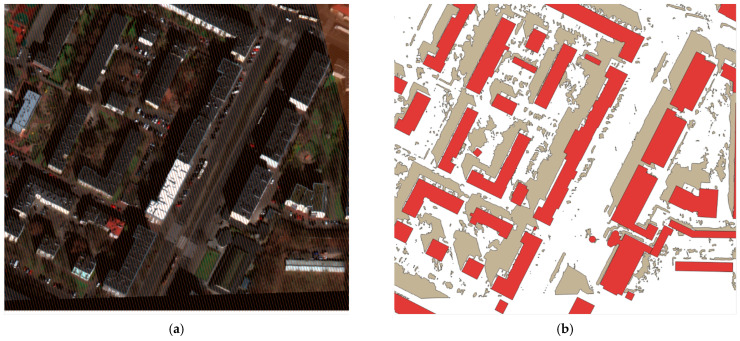
Height estimation process: (**a**) cutting lines (**b**) shadows (brown) and objects (red).

**Figure 10:**
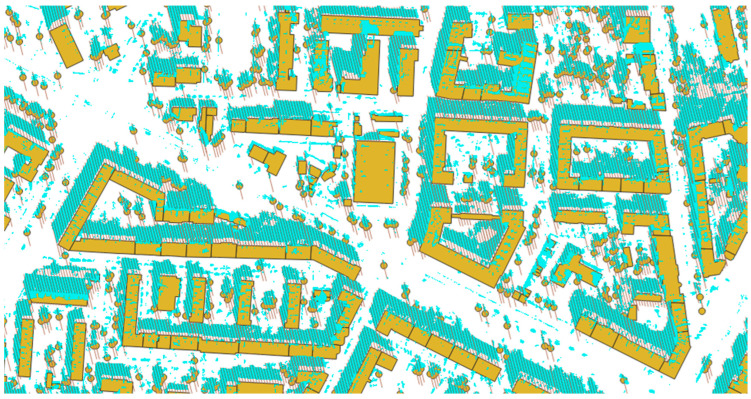
Visualization of shadow–building relationship. Green—shadows, orange—objects.

Next, cutting lines are created according to the reverse azimuth to the azimuth of the sun. This process is illustrated in Figure 9. The next step consists of finding the relationship between objects and shadows through iterative analysis (Figure 10). Additionally, shadows that do not have a corresponding object in the database are analyzed. This is followed by the analysis of objects requiring an individual approach like wind turbines, etc. Then, the length of the shadows is corrected to match the object projection, followed by a process of auto-validation.

### 4.2. Evaluation

In order to evaluate the performance of the entire automated algorithm, the authors decided to compare the results obtained from satellite data with reference LiDAR data for the study area. The publicly available LiDAR point cloud has a density of 12 points per square meter and was acquired on 4 April 2018.

The Mean Absolute Error (MAE) and the Root Mean Square error (RMSE) were chosen as accuracy metrics:

(5) $$RMSE=\;\sqrt{\frac{1}{N}\sum_{i=0}^{N}{\left(h_{i}\;-{\hat{h}}_{i}\right)}^{2}}$$

(6) $$MAE\;=\;\frac{1}{N}\sum_{i=0}^{N}\left|h_{i}\;-{\hat{h}}_{i}\right|$$

where *h_i_* is the estimated height of the object and ${\hat{h}}_{i}$ is the reference height.

The reference height values were obtained by creating a raster that represents the Height Above Ground (HAG) from the LiDAR point cloud. For each vector object, the highest point within its extent was extracted for comparison with the height values obtained from the satellite data.

Initially, it was planned to automate the process of calculating the accuracy parameters by aligning the results from both datasets based on spatial parameters. However, due to the disparity in the acquisition time, this process had to be carried out semi-automatically with manual verification. This is because the Warsaw area is undergoing rapid development, and many new buildings are being constructed that did not exist in 2018 or were still under construction during that period. The preliminary RMSE obtained from the automatic process was 9.7 m.

During the final evaluation of the results, the obtained values of MAE and RMSE were 4.66 and 5.65, respectively.

The next step was the visual evaluation of the results. For this purpose, objects were grouped based on the difference in height between the determined height and the reference height. Four intervals were created: 0–3 m, 3–6 m, 6–10 m, and >10 m. The illustrations below present the images with the results, without considering the exclusion of objects that changed during the period from 2018 to 2020.

**Figure 11:**
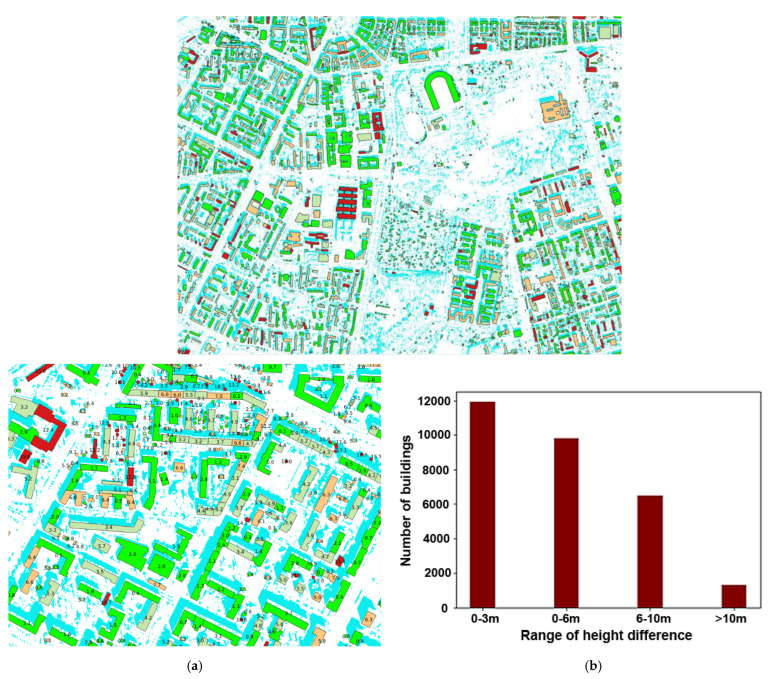
Visualization of differences between reference data and determined data. Green represents differences of 0–3 m, light green of 3–6 m, orange of 6–10 m, and red represents differences above 10 m. Additionally, detected shadows are marked in blue. The **top** image shows a larger view, while the bottom shows (**a**) an image with annotated height estimations differences, and (**b**) the distribution of differences.

The figure (Figure 11) below clearly shows a predominance of the color green, indicating correct height assignments. However, there are a few cases of significant height differences that deserve to be briefly discussed here. As mentioned earlier, some differences above 10 m are due to temporal variations in data acquisition. There are also cases where some objects, such as thin, tall antennas on roofs, are practically impossible to detect with a 0.3 m pixel resolution. Additionally, there are objects where the significant difference is caused by shadow truncation (decreased height) or shadow merging (increased height). These are undoubtedly drawbacks of the developed method. However, in general, the histogram illustrating the filtered data shows a relatively accurate height estimation.

### 4.3. SAMPLE System

The developed algorithm is one of the components of the SAMPLE system (Automatic Air Navigation Obstacles Monitoring System) implemented by Creotech Instruments. SA (Piaseczno, Poland) The SAMPLE system is used for the automatic measurement, processing, visualization, and export of data on aviation obstacles located in a selected area of interest. In addition to a component that utilizes satellite data, there is also the possibility of analyzing point clouds. The system enables the identification of objects that may pose a potential threat to air traffic, especially in airport areas.

**Figure 12:**
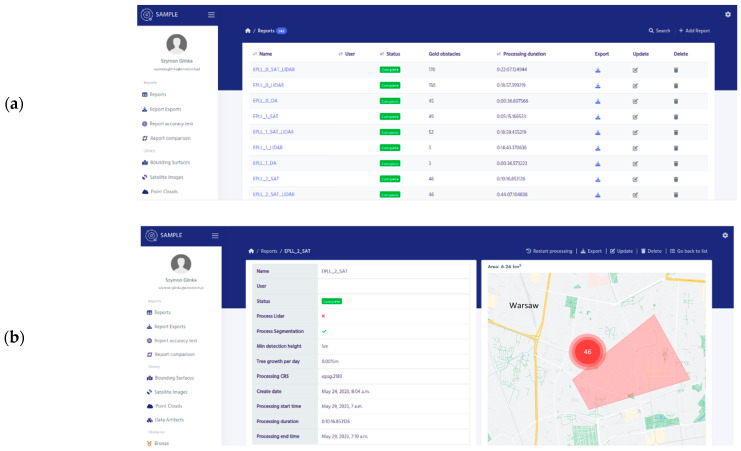
(**a**) Software interface to run and analyze the algorithm. (**b**) Obstacle visualization on map.

Images presenting the software interface are shown below (Figure 12).

## 5. Discussion

At the outset, it should be noted that the obtained results are satisfactory. Obtaining depth from a single image is undoubtedly a significant challenge that may pose certain difficulties in accurately estimating object height. However, it appears that the algorithm, apart from boundary cases (discussed in the previous chapter and further elaborated later), operates at a satisfactory level. The global accuracy of calculating the height of objects in the scene was ±4.66 m. The results obtained are comparable to other results in the literature, in cases where similar quantitative results are available [13,49,53].

The key component for the operation of the entire system is an image with the appropriate parameters and metadata that allow for the spatial identification of the sensor and the photographed area. Through empirical analysis and based on existing cases in the literature, it was noted that the image must also meet certain requirements regarding the aforementioned parameters. This primarily includes the position of the sun, which should not be too low (below 30 degrees) or too high (above 70 degrees). Low sun elevation results in a significant elongation of shadows, which can pose challenges to accurate height estimation. On the other hand, high sun elevation combined with a sensor positioned on the sunny side can lead to an inability to identify shadows in the image as they may be obscured by objects.

One clear advantage of the presented solution is its modularity and the ability to adjust various parameters according to the specific imaging conditions (e.g., threshold). Modifying the models for better efficiency is not a hurdle. Additionally, there are no existing solutions in the subject literature that allow for the height estimation of virtually any object covering the terrain within a given area. It should be noted, of course, that for dense forest areas, height estimation will be limited to forest edges. However, this can still provide valuable information for the entire area, for example by providing the average value.

Another advantage of the solution is its applicability. In addition to its ability to assess the detection of aerial obstacles, it may also be applied in urban planning and mapping of unmapped areas. Height determination may also be used for estimating the population living in a particular area.

There are indeed possibilities to add additional modules, such as utilizing image pairs or SAR (Synthetic Aperture Radar) data. Multi-source verification could potentially generate better results and increase the reliability of the obtained height estimations.

On the other hand, the authors are aware that the developed system has certain limitations. Firstly, it requires images and imagery with specific parameters (as described earlier). Additionally, in densely built-up areas, there may be difficulties in accurately estimating heights due to shadow occlusions on objects or overlapping shadows. Very tall objects and large radial displacements may also introduce errors or make it impossible to determine the heights of objects.

An important aspect that should also be addressed is the accurate and precise georeferencing of the imagery. Precise georeferencing is particularly crucial when using vector objects from open datasets. In cases where there are displacements, the results may be distorted and lead to an unnatural elongation or compression of objects.

Another element is the normalization of satellite images to represent a more realistic RGB image. The normalization technique used can potentially be replaced with style transfer solutions, which could lead to even better results.

## 6. Conclusions

The study indicates that the developed automatic algorithm may be used in preliminary works related to height estimation of buildings. However, for more precise measurements, when the required accuracy is below 1 m, direct measurement methods like geodetic techniques or calculations based on point clouds are necessary. Additionally, during the research, requirements regarding the algorithm’s correct operation were identified, which are related to sensor positioning and sun orientation.

The solution presented in this article allows us to conclude that the analysis of elements from various sources in an image can expand the dimensionality of the obtained data. This, in turn, opens up significant possibilities for spatial analysis using individual imagery.

The authors identified further possibilities for improving the algorithm by creating relationships between objects to assess their mutual influence. They also identified the need for further research to expand the applicability and accuracy of solutions based on single images, as well as attempts to increase the dimensionality of such data.

In the context of describing future work, it is possible to perform a detailed analysis of roofs based on the shadows present on buildings, to generate three-dimensional object models at a defined Level Of Detail (LOD), possibly even LOD 3. This approach may further increase the applicability of the solution, for example, in selecting suitable objects for the installation of solar panels.

## Figures and Tables

**Table 1:** The size of the datasets used for models training.

Task	Train	Validation	Test
Building segmentation	50,000	9000	4520
Shadow segmentation	8000	1200	1200

## Data Availability

The data are not publicly available.
